# Dysregulation of neurodevelopmental regulatory networks in Anorexia Nervosa: an integrated multi-layered omics analysis

**DOI:** 10.3389/fcell.2026.1761785

**Published:** 2026-05-19

**Authors:** Federica Gilardini, Annalaura Sabatucci, Carlo Cifani, Cristina Segura-Garcia, Mariangela Pucci, Marianna Rania, Claudio D’Addario

**Affiliations:** 1 Department of Bioscience and Technology for Food, Agriculture and Environment, University of Teramo, Teramo, Italy; 2 School of Advanced Studies, Center for Neuroscience, University of Camerino, Camerino, Italy; 3 Pharmacology Unit, School of Pharmacy, University of Camerino, Camerino, Italy; 4 Department of Medical and Surgical Sciences, University Magna Graecia of Catanzaro, Catanzaro, Italy; 5 Outpatient Unit for Clinical Research and Treatment of Eating Disorders, University Hospital Renato Dulbecco, Catanzaro, Italy; 6 Department of Clinical Neuroscience, Karolinska Institutet, Stockholm, Sweden

**Keywords:** Anorexia Nervosa, biomarkers, DNA methylation, microbiome, microRNA, multi-omics, neurodevelopment

## Abstract

Anorexia nervosa (AN) is a severe metabo-psychiatric disorder with the highest mortality rate among psychiatric conditions. Its characteristic onset during adolescence suggests that disrupted neurodevelopmental processes during critical periods may contribute to disease pathophysiology. In this exploratory study, we conducted an integrated multi-layered omics analysis to identify molecular alterations affecting neurodevelopmental pathways in individuals with AN. We employed three complementary approaches: DNA methylation sequencing via Reduced Representation Bisulfite Sequencing (RRBS), microRNA (miRNA) expression profiling using panel-based qPCR, and microbiome characterization through 16S rRNA sequencing in a sample of 38 patients with AN and 40 healthy controls. Analyses focused on molecular mechanisms involved in neurodevelopmental processes. RRBS analysis identified methylation differences in neurodevelopmentally relevant genes, particularly *CACNA1C* (voltage-gated calcium channel) and *ZNHIT2* (transcription factor). miRNA profiling revealed extensive dysregulation, with 74 miRNAs showing altered levels in the AN pools. Network analysis highlighted that miR-135 family targets *KCNN3* (SK3 channel involved in neuronal excitability), while miR-374b regulates *IGFBP3* (IGF-1 signaling modulator). Microbiome analysis revealed that 42% of the AN group exhibited dramatic oral dysbiosis characterized by Proteobacteria dominance. Our findings demonstrate coordinated multi-level dysregulation of mechanisms governing neural circuit maturation during adolescence, supporting a neurodevelopmental framework for understanding AN. The convergence of molecular alterations on ion channels and growth factor signaling suggests systems-level perturbations in developmental regulatory mechanisms. The identified miRNAs represent potential biomarkers and therapeutic targets, while microbiome heterogeneity suggests distinct clinical subgroups. While exploratory in nature, this work provides novel insights into AN molecular architecture and generates testable hypotheses for future mechanistic studies incorporating individual-level data essential to validate these candidates and establish robust clinical correlations.

## 1 Introduction

Anorexia nervosa (AN) is a severe psychiatric disorder characterized by persistent restriction of energy intake, intense fear of weight gain, and disturbed body image perception (American Psychiatric Publishing, 2013). With a lifetime prevalence rate up to 4% among females and 0.3% among males and one of the highest mortality rates among psychiatric conditions, AN represents a major public health challenge (van Eeden et al., 2021). Despite decades of research, the neurobiological mechanisms underlying AN remain poorly understood, and effective treatments are limited (Watson and Bulik, 2013; Nagy et al., 2022). A critical feature of AN is its peak onset during adolescence, with incidence rates highest between ages 15 and 19 years, though onset can occur across a broader age range, coinciding with a period of extensive brain maturation including prefrontal cortex development, reward circuit refinement, and synaptic pruning (Casey et al., 2008; Paus et al., 2008; Kaye et al., 2013). This temporal pattern suggests that disrupted neurodevelopmental processes during critical periods may contribute to disease pathophysiology (Casey et al., 2008; Meredith, 2016).

The lack of objective biomarkers represents a major obstacle in AN research and clinical practice (Himmerich and Treasure, 2024). Diagnosis relies entirely on behavioral and psychological criteria, with no molecular tests available for detection, prognosis, or treatment monitoring (American Psychiatric Publishing, 2013). This absence of biomarkers hampers early detection, prevents personalized treatment selection, and limits our understanding of disease mechanisms (Aronson and Ferner, 2017; Glannon, 2022). Moreover, the heterogeneity of AN—with different clinical subtypes, variable illness trajectories, and diverse treatment responses—suggests underlying molecular heterogeneity that remains largely uncharacterized (Kaye et al., 2015). Identification of molecular signatures could enable patient stratification, prediction of treatment outcomes, and development of targeted interventions (Comai et al., 2025).

Multi-level molecular approaches offer unprecedented opportunities to characterize complex disease mechanisms by simultaneously examining multiple layers of molecular regulation (Hasin et al., 2017). Unlike single-platform studies, which provide limited views of biological processes, integrated omics analyses can reveal how different regulatory systems interact to influence phenotypes (Hasin et al., 2017; Morabito et al., 2025). This is particularly relevant for psychiatric disorders, where multiple molecular mechanisms likely converge on neural circuit development and function (Gandal et al., 2018). Here, we focused on three specific molecular layers—DNA methylation, microRNAs, and microbiome—which represent particularly promising targets in the field of eating disorders research.

DNA methylation represents a stable epigenetic modification that regulates gene expression and is responsive to environmental influences (Bird, 2002; Moore et al., 2013). Methylation patterns undergo extensive remodeling during brain development, a critical window for AN onset, where they establish cell-type-specific expression programs and regulate developmental timing (Lister et al., 2013; Jaffe et al., 2016). In AN, aberrant methylation may mediate interactions between genetic vulnerability and environmental factors (Nestler et al., 2016; D’Addario et al., 2017; Thaler and Steiger, 2017; Steiger and Booij, 2020), suggesting that these epigenetic mechanisms could crucially affect disease onset and maintenance (Frieling et al., 2010; Campbell et al., 2011). Moreover, peripheral tissue methylation patterns may serve as accessible biomarkers reflecting disease-relevant processes (Braun et al., 2019; Vismara et al., 2020).

MicroRNAs (miRNAs) are small non-coding RNAs that regulate gene expression post-transcriptionally, with each miRNA potentially controlling hundreds of target genes (Bartel, 2009; Diener et al., 2024). In the developing brain, miRNAs orchestrate critical processes including neurogenesis, neuronal maturation, synapse formation, and activity-dependent circuit refinement (Schratt, 2009; Im and Kenny, 2012). The dynamic regulation of miRNA expression during adolescence makes these molecules particularly relevant for disorders with adolescent onset (Vázquez-Ágredos et al., 2022; Thomas et al., 2023). While dysregulated miRNAs have been documented in various psychiatric conditions, suggesting shared molecular mechanisms across diagnoses (Issler and Chen, 2015; Girella et al., 2025), evidence for AN is still limited, with recent work highlighting convergent epigenetic and miRNA-mediated processes (Voelz et al., 2024; Gilardini et al., 2025). Furthermore, the presence of stable miRNAs in saliva offers opportunities for non-invasive biomarker development (Yoshizawa et al., 2013; Zhang et al., 2024; Mercante et al., 2025).

Finally, the microbiome has emerged as an important modulator of brain function through the microbiota-gut-brain axis, producing neuroactive metabolites and influencing immune function (Cryan et al., 2019). The oral microbiome, the second largest microbial community in humans (Deo and Deshmukh, 2019), may reflect nutritional status, eating behaviors, and metabolic alterations (Kato et al., 2016; Jia et al., 2018; Schamarek et al., 2023). While microbiome research in eating disorders is still emerging, preliminary evidence suggests alterations in microbial communities that could contribute to disease mechanisms or serve as biomarkers (Kleiman et al., 2015; Mack et al., 2016; Landini et al., 2023). The interplay between nutritional restriction, altered eating behaviors, and microbiome composition presents complex bidirectional relationships worthy of investigation (Borgo et al., 2017).

Importantly, these three molecular layers do not function independently. DNA methylation can regulate miRNA gene expression, while miRNAs can target epigenetic machinery components (Moutinho and Esteller, 2017). The microbiome can influence host gene expression through metabolite production, as microbial metabolites such as short-chain fatty acids, and tryptophan derivatives act as signaling molecules that modulate epigenetic mechanisms and transcriptional activity (Stilling et al., 2014; Miro-Blanch and Yanes, 2019; Kim et al., 2024). These interconnections create complex regulatory networks where perturbations at one level may cascade through multiple systems. Integrated analysis of multiple omics layers can therefore reveal convergent pathways and identify genes subject to multi-level dysregulation, providing stronger evidence for pathogenic involvement than single-platform studies (Hasin et al., 2017).

In the present study, we performed comprehensive molecular profiling of individuals suffering from AN compared to healthy controls (HC) using three complementary approaches: RRBS for genome-wide DNA methylation analysis, a panel-based quantitative PCR approach to explore miRNA expression profiling, and 16S rRNA sequencing for microbiome characterization. The analysis focused on identifying molecular patterns affecting neurodevelopmental processes, given the peak adolescent onset of AN and growing evidence for brain structural and functional changes in this disorder (Frank et al., 2013; Titova et al., 2013). The aim of the study was threefold: (1) characterizing DNA methylation patterns in AN with emphasis on neurodevelopmentally relevant genes; (2) comprehensively profiling miRNA expression to identify dysregulated miRNAs and their downstream regulatory networks; (3) examining microbiome composition and diversity.

We selected saliva as the biospecimen for several reasons: non-invasive collection facilitates the study in AN population; saliva contains systemically-derived exosomes that may reflect multi-tissue molecular signatures; the integrated analysis of molecular markers and oral microbiome from the same sample permits examination of host-microbiome interactions; prior studies have validated saliva-derived biomarkers in neuropsychiatric conditions (Kułak-Bejda et al., 2019; Gilardini et al., 2025; Girella et al., 2025; Mercante et al., 2025). We focused on exosomal RNA rather than total cellular RNA because exosomes provide enhanced RNA stability, represent functionally relevant secreted cargo, and partially reduce cellular heterogeneity confounds inherent in whole saliva samples.

## 2 Materials and methods

### 2.1 Study design and participants selection

Participant recruitment took place between April 2022 and May 2023. The clinical cohort consisted of patients attending the Outpatient Unit for Clinical Research and Treatment of Eating Disorders at the University Hospital “Renato Dulbecco” (Catanzaro, Italy). The HC group was recruited from university students through flyers and advertisement.

Inclusion criteria for the clinical group required: (a) confirmed diagnosis of AN through the administration of the Structured Clinical Interview for DSM-5 (SCID-5 CV) and the Eating Disorder Examination (EDE 17.0 D); (b) no ongoing major psychiatric disorders (e.g., major depressive disorder, bipolar disorder, schizophrenia spectrum and other psychotic disorders, substance use disorder) as well as neurodevelopmental and/or intellectual developmental disorders; (c) age ≥14 years old; (d) valid informed consent (legal guardians were informed and asked for the consent in case of minors). AN participants aged ≥14 years were recruited to capture the clinically relevant adolescent population in whom the disorder typically manifests. Inclusion criteria for HCs required: (a) age ≥18 years; (b) no history of eating disorders; (c) Body Mass Index (BMI) within the normal range as defined by World Health Organization guidelines (calculated using the standard formula (kg/m^2^) in both adolescent and adult participants). Exclusion criteria applied to both groups included the presence of chronic systemic diseases, specifically those affecting the immune system, the gastrointestinal tract, or the oral cavity. Variables (including age, sex at birth, educational level, and employment status) and anthropometric measurements (height and weight) were recorded during the clinical examination for patients and using a dedicated questionnaire for HC.

Following the screening process, the final study population comprised 38 individuals in the AN group and 40 in the control group (see Table 1). Median ages were calculated in the two groups using non-parametric Mann Whitney test, accounting for the great variability in the AN group with the presence of outliers, with respect to the more homogeneous healthy control group (Table 1).

**TABLE 1:** Study population characteristics. Demographic and clinical data comparing HC and AN groups.

Characteristics	HC (n. 40)		AN (n. 38)		P value
	Mean ± SD	Median	Mean ± SD	Median	
Age (years)	19.5 ± 0.6	19.0	19.9 ± 7.6	17.5	0.0025
BMI at saliva collection (kg/m^2^)	21.2 ± 2.0	21.1	18.6 ± 2.4 (range: 16.1 ± 1.9–21.9 ± 2.5)	18.3	<0.0001
Weight (kg)	56.6 ± 6.5	55.0	46.8 ± 7.0	46.5	<0.0001
Sex	Female	–	Female	–	–
Age of onset ± SD	–	–	16.7 ± 5.6	–	–
AN clinical features					
Illness duration (years)	–	–	3.2 ± 4.2	–	–
AN subtype					
Restrictive	–	–	23 (61%)	–	–
Binge-purge	–	–	15 (39%)	–	–
Medications					
Antidepressants	–	–	4 (11%)	–	–
Antipsychotics	–	–	2 (5%)	–	–
Antidepressants + Antipsychotics	–	–	17 (45%)	–	–
Other	–	–	3 (8%)	–	–
None	–	–	12 (32%)	–	–

Values presented as mean ± SD and median. Age of onset, range BMI min - BMI max and illness duration applies only to the AN group. AN = Anorexia Nervosa, HC = healthy control.

### 2.2 Ethical approval

The studies involving humans were approved by the Ethical Committee of “Regione Calabria, Area Centro” (identifier: 395/18.11.2021). The studies were conducted in accordance with the local legislation and institutional requirements.

Written informed consent was obtained from all study participants or their legal representatives. Control subjects had previously provided written informed consent for the use of their biological samples/data for research purposes.

### 2.3 Saliva sampling and nucleic acid isolation

Saliva samples were collected exclusively during morning sessions. To preserve sample integrity, participants were instructed to fast, abstain from drinking, smoking, chewing, or performing oral hygiene for at least 1 hour prior to collection. Participants were also asked to report ongoing medical conditions potentially affecting the oral microenvironment, as well as current medications (e.g., antibiotics), and/or the use of dietary supplements such as prebiotics or probiotics, given their potential to interfere with the analysis. Saliva sampling was then performed accordingly. For genomic DNA extraction, a modified salting-out procedure based on the method described by Garbieri et al. was used (Garbieri et al., 2017). Total exosomal RNA was extracted using the Total Exosome Isolation reagent, followed by the Total Exosome RNA and Protein Isolation kit (Invitrogen, Carlsbad, CA, United States), according to the manufacturer’s instructions. The quantity and quality of the extracted DNA were evaluated using a NanoSNAP spectrophotometer (Spex, Metuchen, NJ, United States). Samples demonstrating an A260/280 absorbance ratio of approximately 1.8 were deemed suitable for downstream analysis.

It should be mentioned that saliva contains mixed cell populations (epithelial cells, immune cells, bacteria) and that salivary exosomes derive from multiple cellular sources, potentially influencing methylation and miRNA profiles. Cell-type deconvolution was not performed due to the absence of validated reference libraries for salivary cell types.

### 2.4 Methylation sequencing–RRBS

Genomic DNA was processed using the RRBS protocol. Libraries were prepared following the standard RRBS workflow and indexed for next-generation sequencing (NGS). Sequencing was conducted on the SURFseq5000 platform (GENEMIND BIOSCIENCES Ltd). DNA samples were pooled into four homogeneous groups of 10 subjects each: two pools representing healthy controls (HC) and two pools representing patients diagnosed with Anorexia Nervosa (AN), balanced for age, medication, AN subtype, and body mass index (BMI) (see Supplementary Table S1).

RRBS data were analyzed with R studio 4.4.2 (Posit, Boston, MA, United States). Sequence quality was assessed using FastQC, while pre-processing and trimming of reads were conducted with TrimGalore. Alignment, deduplication, methylation extraction and quality control metrics were performed using Bismark (version v0.24.2). Mapping rates ranged from 34.87% to 54.34% (mean: 42.52% ± 8.48%), consistent with RRBS methodology. Bisulfite conversion efficiency exceeded 99.75% for all samples (mean: 99.77% ± 0.01%), as determined by cytosine methylation rates in non-CpG contexts (CHG and CHH) (see Supplementary Table S2 A for QC metrics). Methylkit package (Akalin et al., 2012) was used for CpG site annotation and coverage filtering (only CpG sites with coverage > 5× were maintained, see Supplementary Table S2 B for the distribution of CpG coverage across pooled libraries). Per-sample CpG coverage before filtering is reported in Supplementary Figure S1. Genomic regions were annotated according to NCBI refseq_id annotation.

The evaluation of methylation patterns on protein-coding genes was performed as follows: starting from the 13,378 CpG annotated positions detected by RRBS, we filtered out all the positions containing at least an NA value in the four groups (2 HC–HC1, HC2, and 2 AN–AN1, AN2) and all the positions corresponding to “unknown” in region or refseq_id. These positions are located in long noncoding (lnc) regions of the genome. In this way, we had only 148 positions left.

Among them, we filtered out 32 positions with 0% methylation in all four groups; seven positions with 100% methylation in all four groups; 25 belonging to uncharacterized regions. In this way, we had only 73 positions left, corresponding to exons, introns, and promoters (up to 2 kb from TSS) of protein-coding genes.

### 2.5 miRNA panel analysis

miRNA expression profiling was performed using the Human Panel 1, V6 miRCURY LNA miRNA miRNome PCR Panel (YAHS-510ZF, Qiagen), which enables SYBR® Green-based quantification of 384 most abundantly expressed and best characterized human miRNA sequences. This panel is optimized for focused miRNome analysis and includes miRNAs selected for their biological relevance across a range of physiological and pathological conditions.

Exosomal RNA samples were pooled into nine groups: three pools of AN individuals (n = 10 per pool) and six pools of HC individuals (n = 4–10 per pool) (see Supplementary Table S3 for clinical and demographic profiles of the pooled libraries). This design provides increased biological replicates in the control group, enhancing statistical robustness of the reference healthy signature.

Reverse transcription was carried out using the miRCURY LNA RT Kit, according to Qiagen’s recommended procedure. Quantitative real-time PCR was then performed using the miRCURY LNA SYBR® Green PCR Kit, adhering to the standard cycling conditions and data analysis workflow provided by Qiagen.

To focus on robustly expressed targets, only miRNAs with Ct values <35 were included, potentially avoiding the inclusion of noise from low-abundance targets and ensuring reliable quantification. Data quality was evaluated based on hemolysis assessment and call rate metrics integrated into the Qiagen workflow. Hemolysis was monitored using the ΔCt ratio of miR-23a-3p to miR-451-5p, which showed no evidence of significant red blood cell contained in the analyzed pools. The detection consistency was evaluated through the call rate, which averaged approximately 58% for HC and 64% for AN pools. These metrics confirmed that the salivary exosomal RNA provided a sufficiently robust profile for the subsequent analysis.

Raw Ct values were normalized using the reference miRNAs included in the panel (hsa-SNORD38B and hsa-SNORD49A). Relative expression levels were calculated using the comparative Ct method (ΔΔCt), following the automated data analysis and quality control workflow provided by Qiagen. Log_2_ fold change (log_2_FC) values were used for downstream analysis. To ensure biological relevance and account for the pooled design, a stringent threshold of |log_2_FC| > 2 was applied for the identification of altered expressed miRNA patterns within the pools.

miRNA network analysis was conducted in R studio 4.4.2 (Posit, Boston, MA, United States). To construct the network, only miRNAs predicted to target genes associated with Anorexia Nervosa (retrieved from MalaCards Human Disease Database) and showing a prediction score >90 in miRDB were considered. Target prediction was done using multiMiR R package (Quast et al., 2013; Ru et al., 2014).

### 2.6 Microbiome analysis

Salivary microbiome of 40 healthy control (HC) and 33 Anorexia Nervosa (AN) samples was studied. 30 μL DNA samples, 25 ng/μL were utilized for 16S rRNA sequencing (Wellmicro®, Italy). The sequences of V3-V4 amplified regions of 16S RNA were analyzed with QIIME2 amplicon suite 2024.10 (Bolyen et al., 2019). A denoising step on fastq sequences to remove low quality regions was performed with DADA2 plugin (Callahan et al., 2016). A tree for phylogenetic difference analysis was built using the pipeline based on the mafft multiple alignment program. Alpha and Beta diversity analyses were performed on aligned sequences to assess respectively community richness and similarity. Alpha diversity was assessed by evaluating Faith’s Phylogenetic Diversity and Evenness vector distances. Beta diversity was evaluated by Principal Component Analysis (PCoA). We evaluated unweighted and weighted UniFrac distances to have a qualitative and quantitative measure of community dissimilarity, considering phylogenetic relationships between the features. For taxonomic classification of amplicon sequencing, the Silva (Quast et al., 2013) pre-built full length weighted classifier (v138) was utilized. Differential abundance of Operational Taxonomic Units (OTUs) was calculated with the ancom-BC tool in QIIME2.

## 3 Results

### 3.1 Methylation sequencing - RRBS

**FIGURE 1:**
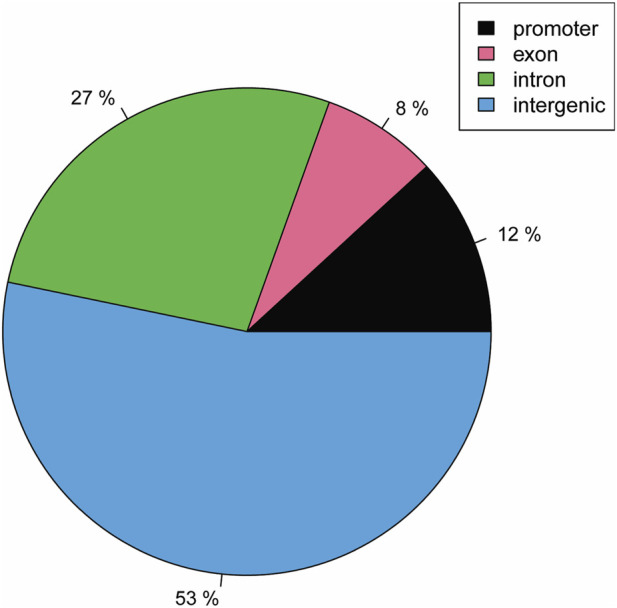
Pie plot of genomic distribution of differently methylated CpG sites (>25%) in saliva from Anorexia Nervosa (AN) and Healthy Control (HC) groups samples analyzed by RRBS.

We first considered the CpG sites with overall differences in methylation >25% and q-value ≤0.01 between the AN and HC groups. As shown in Figure 1, when looking at the methylation percentage difference per genomic regions, 53% of the total sites are in intergenic regions, while promoter regions (up to 2 Kb upstream the TSS), which are known to be crucial for gene transcription regulation (Stefansson et al., 2024), account for 12% of the overall methylation differences. We then narrowed the analysis to CpG sites of protein-coding genes (see material and methods). 11 positions resulted in significantly different methylation percentages in the AN groups with respect to the HC groups (see Supplementary Table S4): 3 in *OR4F3* (Olfactory receptor 4F3/4F16/4F29) promoter; 3 in pseudogene *BAGE2* (B melanoma antigen-*BAGE* family 2 member) intron and 1 respectively in *ZNHIT2* (Zinc finger HIT domain-containing protein 2) promoter; *CACNA1C* (Voltage-dependent L-type calcium channel subunit alpha-1C) intron; pseudogene *ROCK1* (Rho associated coiled-coil containing protein kinase 1) intron; *SENP5* (SUMO specific peptidase 5) intron; and *GAGE2A* (G antigen 2A) intron. Among them, data in literature indicate that two genes are possibly related to AN *ZNHIT2*, a transcription factor that has been associated with stress, energy and nutrition-related pathways (Cloutier et al., 2017) and the *CACNA1C* calcium channel with variants previously related to psychiatric disorder risk (Wang et al., 2023).

### 3.2 miRNA panel analysis

**FIGURE 2:**
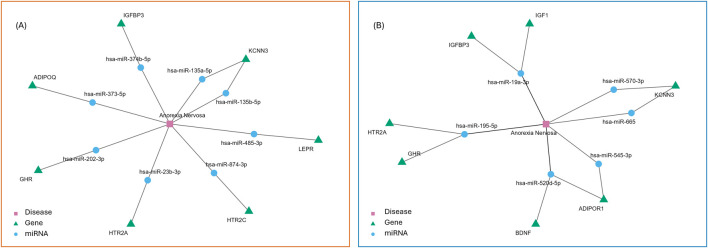
miRNA-gene-disease network derived from differentially expressed miRNAs. Blue circles represent miRNAs, green triangles represent genes, the red square represents disease, and edges indicate predicted interactions. The red framed network **(A)** corresponds to upregulated miRNAs, blue framed network **(B)** corresponds to downregulated miRNAs.

A total of 74 miRNAs were found to be expressed at different levels in AN versus HC pools. Of these, 11 miRNAs exhibited downregulation with log_2_FC ≤ −2, whereas 63 miRNAs were upregulated with log_2_FC ≥ 2. The complete list of these dysregulated miRNAs, together with their fold change values, is reported in Supplementary Table S5, which provides a detailed overview of the expression patterns observed in the analyzed study cohort. Subsequent construction of the miRNA-gene-disease network, as described in the Material and Methods section, revealed specific connections between deregulated miRNAs and their target genes. In particular, six downregulated miRNAs were connected to seven distinct genes, and eight upregulated miRNAs were associated with seven genes (Figure 2). Within this network, the analysis highlighted interactions involving genes critical for neuronal excitability and developmental signaling, processes known to be dysregulated in AN pathophysiology. Specifically, the miR-135 family (miR-135a-5p and miR-135b-5p) displayed a direct connection to potassium calcium-activated channel subfamily N member 3 (*KCNN3*), the gene encoding the small-conductance calcium-activated potassium channel 3 (SK3) involved in the regulation of firing patterns and neuronal excitability (Stocker et al., 1999). Concurrently, miR-374b-5p was linked to insulin-like growth factor binding protein 3 (*IGFBP3*), a key modulator of the insulin-like growth factor 1 (IGF-1) signaling pathway which governs synaptic and structural maturation (Dyer et al., 2016; Allard and Duan, 2018; Okuyama et al., 2021).

### 3.3 Microbiome analysis

**FIGURE 3:**
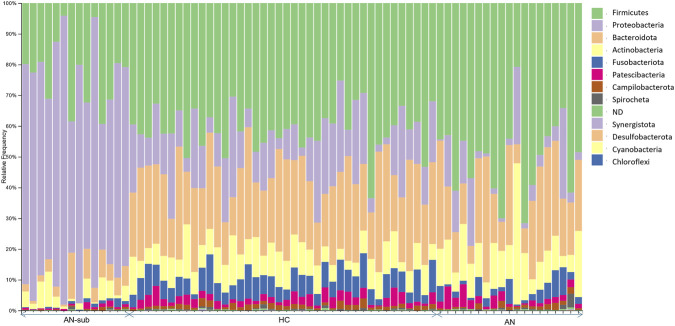
Bar plot of relative phylum abundance in salivary microbiota of healthy controls (HC), Anorexia Nervosa (AN) samples and of Anorexia nervosa subgroup (AN-sub) with peculiar phylum distribution.

**FIGURE 4:**
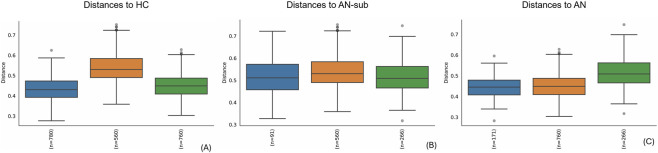
Unweighted UniFrac distances. **(A)** AN (green) and AN-sub (orange) groups to HC (blue); **(B)** AN (green) and HC (orange) groups to AN-sub (blue); **(C)** AN-sub (green) and HC (orange) groups to AN (blue).

**FIGURE 5:**
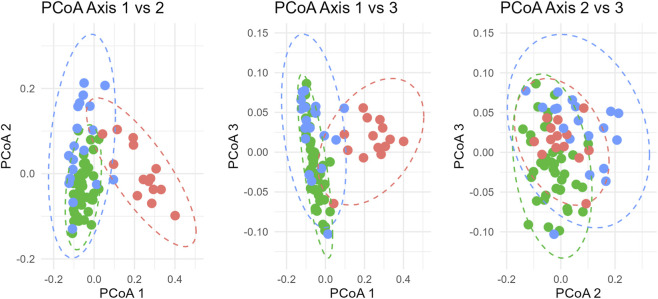
Weighted UniFrac distance PCoA representation according to three different projections of the first three components of health control (green), AN (cyan) and AN-sub (red) samples.

**FIGURE 6:**
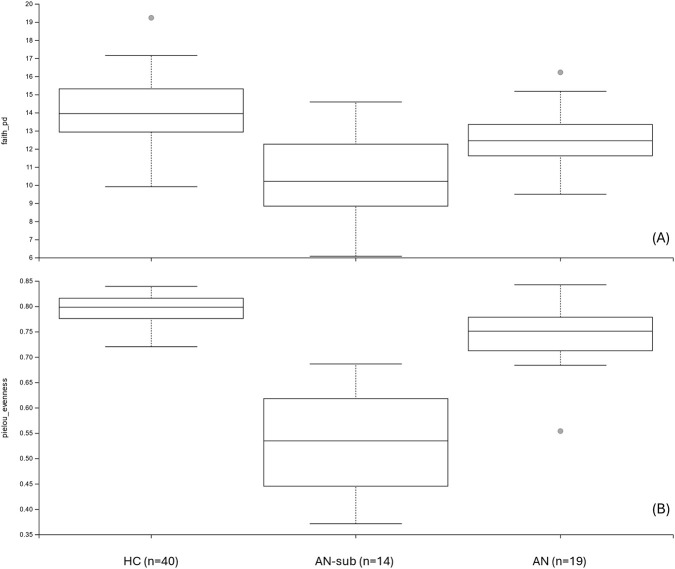
Alpha diversity analysis of the three different groups of healthy controls (HC), Anorexia Nervosa (AN) and Anorexia Nervosa subgroup (AN-sub). **(A)** Faith Phylogenetic Distance (PD); **(B)** Pielou Evenness.

**FIGURE 7:**
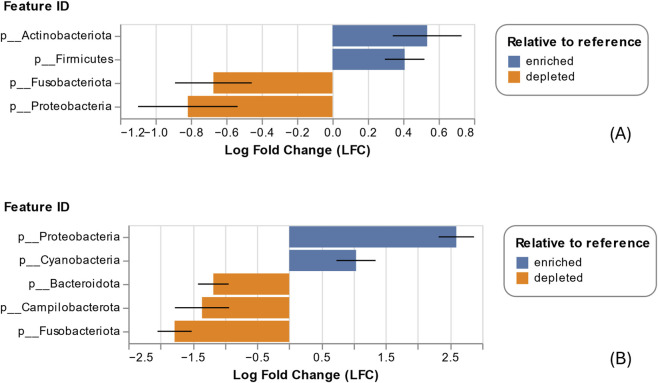
Abundance differences (log fold change) at phylum level. **(A)** AN vs. HC; **(B)** AN-sub vs. HC.

The microbiome analysis was conducted on individual DNA samples from saliva (see materials and methods section). The taxonomic analysis of the sequenced DNA revealed that 99.979% of total sequences were bacterial, indicating high purity of the samples. The analysis of phylum distribution of all samples revealed the presence of a sub-group of 14 samples in the AN group with a peculiar phylum distribution (see Figure 3) with a prevalence of Proteobacteria (41% up to 94% relative frequency in each sample) while in the HC group and on the other 19 AN samples, the most abundant phylum were found to be Firmicutes. Individual-level microbiome analysis of AN sub-group did not identify statistically significant associations with any clinical parameter examined, including AN subtype, purging behaviours in the last 28 days, illness duration, age, BMI at sample collection date and minimum BMI (data not shown); however, these findings should be interpreted with caution as the study was not formally powered to detect subgroup-specific effects. Deepening the analysis, we were able to classify the most abundant OTUs within this sub-group (see Table 2): beta diversity analysis showed that AN-sub samples form a distinct cluster, separated from the main AN and from the HC groups clustered apart both qualitatively and quantitatively. The unweighted UniFrac distances (Figure 4), indicate that all groups are significantly different. The most significant distance is between AN-sub and HC group. Pseudo-F values of pairwise permanova test are reported in Supplementary Table S6. In Figure 5 the PCoA of weighted UniFrac distances is reported, a quantitative measure of group dissimilarity taking into account phylogenetic relationships, clearly showing the different distribution of AN-sub samples with respect to the other groups. We then considered alpha diversity and evaluated both Faith phylogenetic distance (PD) and evenness vector (Figure 6; Supplementary Table S7). All distances were found to be significant except PD diversity between AN vs. AN-sub groups (p = 0.012) (See Supplementary Table S7 for Kruskal–Wallis test results). We then evaluated the differential abundance of different OTUs considering a significance threshold of p < 0.05. At the phylum level, both AN and AN-sub exhibited a depletion of Fusobacteriota with respect to HC group while Proteobacteriota were depleted in AN group and enriched in AN-sub group (see Figure 7).

**TABLE 2:** Most abundant OTUs (S: species; G: genus; F: family) in AN-sub group.

Sample	Main group/species	%
1	S: *Pseudomonas reactans*	70.692%
2	S: *Pseudomonas reactans*	49.720%
3	S: *Pseudomonas reactans*	47.431%
4	G: *Pseudomonas*	45.224%
5	G: *Stenotrophomonas*	78.240%
6	S: *Serratia nematodiphila*	93.657%
7	G: *Stenotrophomonas*	24.774%
8	F: *Enterobacteriaceae*	46.670%
9	G: *Stenotrophomonas*	46.696%
10	G: *Stenotrophomonas*	65.002%
11	S: *Pseudomonas reactans*	15.719%
12	S: *Pseudomonas reactans*	50.614%
13	G: *Stenotrophomonas*	67.643%
14	G: *Stenotrophomonas*	62.751%

## 4 Discussion

This exploratory integrated multi-layered omics analysis reveals a complex molecular landscape highlighting extensive miRNA dysregulation, specific epigenetic alterations in neurodevelopmentally important genes, and striking microbiome profiling in a substantial patient subgroup. Present findings suggest group-level associations between AN pathophysiology and molecular alterations in pathways implicated in brain development and functions, particularly those governing ion channel maturation and growth factor signaling during adolescence.

The interpretation of the molecular findings should consider that the pooled sample approach precludes direct examination of how individual clinical variables may modulate the observed molecular signatures. However, the individual-level microbiome analysis did not reveal significant associations between any of these parameters and microbiota diversity, suggesting that the identified alterations may reflect a general AN pathophysiology rather than subgroup-specific phenomena.

Notably, the consistency of these molecular signatures across our cohort, spanning a wide age range, suggests that these alterations might reflect durable biological imprints of neurodevelopmental changes occurring during the adolescence, and not an age-dependent epigenetic drift. The persistence into adulthood in fact is consistent with the neurodevelopmental hypothesis and aligns with the adolescent peak of AN onset outlined in the introduction section.

### 4.1 DNA methylation: targeted alterations in neurodevelopmental genes

The RRBS analysis identified distinct methylation patterns in specific genes relevant to neurodevelopment, whereas global methylation profiles did not clearly discriminate between cases and controls. The observation that only 12% of methylation differences occurred in promoter regions, with the majority in intronic and intergenic sequences, is consistent with modern understanding of gene regulation (Stadler et al., 2011; Ziller et al., 2013). These non-promoter elements often contain enhancers and other regulatory sequences critical for developmental and cell-type-specific expression (Nord and West, 2019). The distribution of methylation changes suggests that AN may be associated with alterations in developmental enhancers rather than constitutive transcriptional control.

These observations align with the growing body of evidence indicating that AN is characterized by broad, state-dependent epigenetic remodeling. Specifically, systematic reviews of epigenome-wide association studies (EWAS) have identified extensive differential methylation in genes regulating metabolism and psychiatric status, suggesting that these signatures may serve as biomarkers for specific disease stages (Käver et al., 2024). Supporting the dynamic nature of these changes, Steiger et al. (Steiger et al., 2023) identified over 200 altered methylation sites in patients during the acute phase of AN but found a significant normalization of these patterns in long-term remitted individuals, suggesting that the epigenetic landscape in AN reflects the physiological state of malnutrition rather than permanent genetic scars. While clinical research has focused on the hypomethylation of energy-balance regulators like Leptin promoter as a tracker for treatment success (Neyazi et al., 2019), our identification of methylation shifts in neurodevelopmental candidates such as *CACNA1C* and *ZNHIT2* suggests that the epigenetic response observed in the analyzed pools also involves more stable circuits related to activity-dependent gene transcription and stress regulation.

Among the genes showing altered methylation, *CACNA1C* emerged as particularly notable. This gene encodes the voltage-gated L-type calcium channel Cav1.2, which plays essential roles in activity-dependent gene transcription and neuronal development (Greer and Greenberg, 2008; Bhat et al., 2012; Herold et al., 2023). L-type calcium channels couple neuronal activity patterns to transcriptional programs, enabling experience-dependent circuit refinement during adolescence (West and Greenberg, 2011; Baker et al., 2023). *CACNA1C* represents one of the most consistently replicated risk genes across psychiatric disorders, with genome-wide association studies linking it to schizophrenia, bipolar disorder, major depression, and autism spectrum disorders (Green et al., 2009; Bhat et al., 2012; Smoller et al., 2013; Kabir et al., 2017; Herold et al., 2023). This remarkable pleiotropy suggests that *CACNA1C* disruption may be associated with fundamental neurodevelopmental processes that, when perturbed, can manifest as different psychiatric phenotypes depending on additional genetic and environmental factors (Dedic et al., 2017). The identification of *CACNA1C* methylation variability here provided for AN extends the range of psychiatric disorders associated with this gene and supports models proposing shared neurodevelopmental mechanisms across diagnostic boundaries (Owen and O’Donovan, 2017). The methylation change we observed occurred in an intronic region of *CACNA1C* rather than the promoter. While this may seem less significant, intronic regions frequently contain regulatory elements affecting alternative splicing, tissue-specific expression, and developmental regulation (Chorev and Carmel, 2012). The functional consequences of this specific methylation change require experimental validation, but the finding suggests potential for altered *CACNA1C* expression or isoform usage in AN. Given the central role of calcium signaling in neurodevelopment and the pleiotropic psychiatric associations of *CACNA1C*, this represents a compelling candidate for functional follow-up studies (Rodan et al., 2021; Wang et al., 2023). Of note, *CACNA1C* was recently predicted to have deleterious variants potentially associated with syndromic forms of AN (Ceccarini et al., 2021).

*ZNHIT2*, a gene that encodes a zinc finger transcription factor, also exhibited altered promoter methylation levels among groups. *ZNHIT2* has been associated with stress, energy balance and nutrition-related pathways, helping assemble the U5 small nuclear ribonucleoprotein (snRNP), a key spliceosome component, relevant to brain development and function (Cloutier et al., 2017; Nava et al., 2025). The methylation change in *ZNHIT2* could represent environmentally-induced epigenetic alterations potentially associated with lasting changes in stress-responsive gene regulation (Szyf, 2013). Such developmental epigenetic patterns could be associated with the persistence of AN even after behavioral symptoms improvement, as altered epigenetic states may be related to abnormal stress responses and metabolic regulation (Steiger and Booij, 2020).

AN encompasses different subtypes (restricting versus binge-purge), varying illness durations, diverse genetic backgrounds, and different treatment histories (Resmark et al., 2019; Watson et al., 2022; Abber et al., 2025). This clinical heterogeneity may translate into molecular heterogeneity. While this heterogeneity presents challenges for identifying universal biomarkers, it may ultimately inform personalized medicine approaches if molecular subtypes can be reliably characterized and correlated with clinical features or treatment responses (Breithaupt et al., 2024; Samodova et al., 2024).

### 4.2 Extensive microRNA dysregulation: post-transcriptional control of neurodevelopment

The most striking finding of our study was the extensive dysregulation of miRNAs expression observed in the AN pools compared to controls. This magnitude of dysregulation contrasts sharply with the modest methylation changes and represents the most robust molecular signature we identified. The bidirectional nature of miRNA changes—with many showing increased expression while others showed decreased expression—indicates complex regulatory remodeling rather than simple unidirectional responses to illness or starvation.

Our results represent a significant expansion of the current knowledge regarding miRNA profiles in AN, a field where clinical research remains notably limited (Voelz et al., 2024). To date, the available evidence has been restricted to two studies: a pre-clinical investigation identifying miR-340 as a key placental regulator involved in the prenatal programming of AN-like vulnerability in a mouse model and our recent targeted analysis of the endocannabinoid system (ECS) (Schroeder et al., 2018; Gilardini et al., 2025). In that previous work, we reported miRNA dysregulation targeting specific ECS genes in the AN group, coupled with promoter methylation alterations, suggesting an ECS impairment in eating disorders. By providing a systemic and comprehensive analysis of salivary miRNAs in human patients, our current findings offer a much-needed clinical expansion to this restricted evidence base. The extensive dysregulation observed here suggests associations with fundamental alterations in a broader range of post-transcriptional regulatory networks. Given that miRNAs are key regulators of gene expression, and each miRNA can impact hundreds of targets, the widespread dysregulation we observed could potentially be associated with altered cellular function (Ebert and Sharp, 2012; Bartel, 2018). In the context of neurodevelopment, where miRNAs orchestrate processes including neurogenesis, neuronal differentiation, synapse formation, and circuit refinement, such extensive dysregulation during adolescence could potentially be associated with aberrant developmental trajectories (Follert et al., 2014; Rajman and Schratt, 2017).

Network analysis revealed that dysregulated miRNAs converge on specific genes and pathways of neurodevelopmental importance. Among the upregulated miRNAs, the miR-135 family (miR-135a-5p and miR-135b-5p) showed particularly strong network connections to *KCNN3*, which encodes the SK3 channel. SK3 channels regulate neuronal excitability by contributing to afterhyperpolarization following action potentials (Stocker et al., 1999; Adelman, 2015). By controlling the degree and duration of afterhyperpolarization, SK channels influence firing patterns, spike frequency adaptation, and synaptic integration (Stocker et al., 1999). During development, SK channel expression undergoes dynamic regulation to establish neuronal excitability as circuits mature (Cingolani et al., 2002).

From a behavioral perspective, SK3 channels have been specifically implicated in fear and anxiety circuits. Rodent studies demonstrate that SK channel modulation affects fear conditioning, extinction, and anxiety-like behaviors (Criado-Marrero et al., 2014). SK3 expression is detected in limbic regions critical for emotional processing, specifically the amygdalo-hippocampal area, dentate gyrus, and amygdala nuclei (Tacconi et al., 2001). Furthermore, dense SK3 expression in the lateral septum and locus coeruleus support their role in anxiety regulation (Borodovitsyna et al., 2020; Iftikhar et al., 2020). Notably, some of these same regions have been documented to show structural alterations in AN, such as reduced volume in the hippocampus-amygdala and specific amygdala nuclei (Giordano et al., 2001; Kappou et al., 2021; Wronski et al., 2023). Consequently, based on predicted regulatory relationships, the upregulation of miR-135a/b observed in the AN group would be predicted to suppress *KCNN3* expression, potentially affecting SK3 channel function, and increasing neuronal excitability. This molecular mechanism could be hypothesized to contribute to the heightened anxiety characteristic of AN, particularly the exaggerated fear responses to food and weight gain (Di Lodovico et al., 2024). Our cross-sectional design cannot establish whether miRNA alterations precede, accompany, or follow the development of anxiety symptoms.

Critically, if miR-135 upregulation occurs during adolescence, when fear and anxiety circuits undergo developmental refinement, it could potentially be associated with lasting alterations in circuit properties. Adolescence represents a critical period for establishing appropriate excitatory-inhibitory balance and anxiety set points through activity-dependent mechanisms (Pattwell et al., 2012; Gee et al., 2013; Baker et al., 2014). This is particularly relevant for the miR-135 family, which we found upregulated in the AN pools: miR-135 has been identified as essential for chronic stress resilience and intact serotonergic activity in anxiety circuits (Issler et al., 2014), suggesting that its developmental dysregulation could be associated with the persistent anxiety symptoms observed in AN even after weight restoration. This neurodevelopmental framework provides one potential explanation for why AN typically emerges during adolescence (Herpertz-Dahlmann, 2015) and why anxiety symptoms often persist as a stable trait even after successful weight restoration and normalization of eating behaviors (Kaye et al., 2004). However, establishing causal relationships between peripheral miRNA changes and brain circuit alterations requires longitudinal and functional validation studies.

Another prominently dysregulated miRNA, miR-374b-5p, showed network connections to *IGFBP3*, a key modulator of IGF-1 bioavailability and activity which plays a critical role in growth factor signaling (Martín et al., 2021; Okuyama et al., 2021). IGF-1 signaling is pivotal for integrating neuronal development with synaptic plasticity and metabolic regulation (Dyer et al., 2016; Allard and Duan, 2018; Okuyama et al., 2021), with decreased levels consistently reported in clinical studies conducted in individuals with AN (Gianotti et al., 2002; Fazeli et al., 2019; Wu et al., 2024).

From a neurodevelopmental perspective, alterations in IGF-1 signaling during the sensitive window of adolescent brain maturation could potentially have significant consequences. Prolonged perturbation of IGF-1 signaling during this critical period could be associated with the structural brain alterations documented in AN, including reduced gray matter volume and altered white matter integrity (Titova et al., 2013; Seitz et al., 2016). This may create a potential association: reduced IGF-1 due to dietary restriction may be linked to impaired neurodevelopment, potentially contributing to brain changes that are associated with cognitive rigidity and altered reward processing, which could ultimately reinforce the pathological eating behavior (Kaye et al., 2013). miRNA-mediated dysregulation of growth factor signaling could amplify these effects or impair compensatory responses. Again, the directionality of these relationships, whether miRNA changes drive metabolic alterations, result from them, or represent parallel processes, cannot be determined from our cross-sectional data.

Of particular note is how these miRNAs converge on two systems critical for neurodevelopment: the *KCNN3* ion channel and the *IGFBP3* growth factor pathway. These findings suggest that AN could be associated with coordinated alterations in multiple developmental regulatory pathways rather than isolated molecular changes. Furthermore, the integration of our miRNA findings with the *CACNA1C* methylation results suggests multi-level dysregulation of calcium signaling systems, including both voltage-gated calcium channels and calcium-activated potassium channels. This systems-level alteration in mechanisms governing neuronal excitability and activity-dependent development emerges as the unifying theme of our multi-layered omics analysis.

### 4.3 Oral microbiome dysbiosis: a novel an subgroup

In accordance with a recent study (Vignal et al., 2025), our analysis revealed significant differences in oral microbiome composition of AN patients with respect to HC controls. Even though our cohort differs in terms of age and characteristics, the consistent observation of significant shifts in both alpha and beta diversity across independent populations validates oral dysbiosis as a robust and reproducible biological signature of AN.

In addition, our microbiome analysis revealed an unexpected and striking finding: approximately 42% of participants in the AN group (14 of 33) exhibited dramatic alterations in oral microbiome composition. Proteobacteria dominance and substantial enrichment of specific bacterial genera including *Pseudomonas, Stenotrophomonas, Serratia*, and *Enterobacteriaceae* marked a different microbial profile with respect to HC, and within the AN group, representing a previously unrecognized heterogeneity within the AN population.

After excluding potential individual factors, we considered whether this striking pattern might arise from technical contamination during sample collection or processing. However, several critical observations argue against contamination as the primary explanation. Most importantly, samples from participants with and without this dysbiosis as well as controls, were collected and processed on the same days using identical protocols, reagents, and personnel. If contamination had occurred during processing, we would expect to see batch effects correlating with processing date, which we did not observe. The random distribution of dysbiotic samples across different collection and processing batches strongly argues against technical artifacts. What, then, might explain the dramatic microbiome alterations in this AN subgroup? Several disease-related mechanisms warrant consideration. First, severe dietary restriction in AN, produces profound changes in nutritional intake, including macronutrient and micronutrient deficiencies (Affenito et al., 2002; Misra et al., 2006; Setnick, 2010; Chiurazzi et al., 2017; Hanachi et al., 2019; Pereira et al., 2022; Jenkins et al., 2024). The oral microbiome is highly responsive to dietary composition, with different bacterial taxa showing preferences for specific nutrients (Lassalle et al., 2018). The extremely restricted and often monotonous diet within the AN clinical framework could create selective pressures favoring certain bacterial species while suppressing others. The types of bacteria enriched in the dysbiotic subgroup—many of which are metabolically versatile organisms capable of utilizing diverse nutrient sources—might represent species better adapted to the specific disruption of the nutritional environment. Second, malnutrition-associated immune dysfunction could permit colonization by opportunistic organisms that would normally be controlled by immune surveillance. Alterations in both innate and adaptive immunity, including changes in neutrophil function, natural killer cell activity, and cytokine production have been frequently reported in this disorder (Marcos, 2000; Maunder et al., 2023). Reduced immune competence could allow environmental bacteria to colonize and persist in the oral cavity when they would typically be excluded. The enrichment of organisms such as *Pseudomonas* and *Stenotrophomonas*—known opportunistic pathogens—is consistent with this immunocompromise hypothesis. Third, alterations in salivary composition secondary to malnutrition could create environmental conditions favoring specific bacterial communities. Saliva provides nutrients, antimicrobial proteins, and buffering capacity that shape microbiome composition (Wade, 2013; Lynge Pedersen and Belstrøm, 2019). Malnutrition affects salivary gland function, potentially altering the composition and flow rate of saliva (Johansson et al., 2012; Huang et al., 2022). Changes in salivary pH, antimicrobial protein concentrations, or nutrient availability could fundamentally reshape the oral microbial ecosystem. Some studies have documented reduced salivary flow rates in AN, which could impair mechanical clearance of bacteria and alter the oral environment (Dynesen et al., 2008; Niederau et al., 2025). Fourth, stress-induced alterations in the oral environment might contribute to dysbiosis. AN is associated with chronic psychological stress and persistently elevated cortisol levels (Lawson et al., 2009; Calcaterra et al., 2024). Stress hormones can affect immune function, inflammation, and tissue physiology in ways that could influence microbial communities (Bailey et al., 2011; Alotiby, 2024). The autonomic nervous system, which is frequently dysregulated in AN, also modulates salivary secretion and composition, providing another pathway through which stress could affect the oral microbiome (Mazurak et al., 2011; Proctor, 2016).

The identification of two distinct microbiome profiles within the AN population—one of which showing dramatic dysbiosis—suggests important clinical heterogeneity. This raises several intriguing questions: does the dysbiotic subgroup represent a more severe form of illness? Do these patients have different illness trajectories or treatment responses? Are there genetic or environmental factors predisposing certain individuals to microbiome alterations? More detailed clinical phenotyping might reveal subtle differences in eating behaviors, nutritional intake patterns, medical complications, or psychological features.

Our findings on oral dysbiosis parallel the alterations in the gut microbiota of AN patients, where systematic studies have confirmed a state of generalized dysbiosis (Seitz et al., 2016; Garcia and Gutierrez, 2023). Causality relations have also been inferred between gut microbiome alteration and AN pathogenesis (Fan et al., 2023), and a link between gut microbiota alteration and epigenetic factors in AN has recently been proposed (Korten et al., 2025). The gut-brain axis literature demonstrates that microbiome composition can influence brain function through multiple pathways including production of neuroactive metabolites (short-chain fatty acids, neurotransmitter precursors), modulation of systemic inflammation, and effects on gut-derived hormones (Cryan et al., 2019). While most research has focused on the gut microbiome, the oral microbiome also produces metabolites that enter the bloodstream and could theoretically influence brain function (Bostanci and Belibasakis, 2012; Wang et al., 2024).

From a mechanistic perspective, the microbiome alterations we observed could be purely consequences of AN pathophysiology, or they could actively contribute to disease maintenance through microbiota-brain interactions. Specific to neurodevelopment, emerging evidence suggests that microbiome alterations during critical periods can affect brain maturation. Animal studies demonstrate that antibiotic-induced microbiome disruption during adolescence can produce lasting changes in brain structure and behavior (Desbonnet et al., 2015). If the oral microbiome dysbiosis we observed occurs during the adolescent period when AN typically develops, it could potentially contribute to or amplify neurodevelopmental perturbations. However, this remains speculative and would require longitudinal studies examining microbiome composition before, during, and after illness onset.

The consistent finding of Fusobacteriota depletion across both AN subgroups (dysbiotic and non-dysbiotic) compared to controls represents another interesting observation. Fusobacteria are members of the oral microbiome with roles in oral ecology and biofilm formation (Brennan and Garrett, 2019). Their depletion in AN represents a subtle, shared alteration across all AN patients, independent of the dramatic dysbiosis seen in the subgroup. This consistent depletion might be more directly related to disease-specific factors such as dietary composition or oral hygiene behaviors.

### 4.4 Clinical and therapeutic implications

Our findings have several potential clinical and therapeutic implications. The robust miRNA dysregulation could serve as a biomarker for diagnosis, prognosis, or treatment response monitoring. Saliva non-invasive collection and stability make it ideal for tracking miRNA signatures in at-risk adolescents or monitoring recovery (Luoni and Riva, 2016; Zhang et al., 2016; Khoodoruth et al., 2024).

The molecular targets identified represent potential therapeutic entry points. SK channels (encoded by *KCNN3*) can be modulated by small molecules investigated for neuropsychiatric applications (Dolga and Culmsee, 2012). If miR-135-mediated suppression of *KCNN3* is confirmed to contribute to anxiety phenotypes through functional studies, pharmacological SK channel activation may reduce anxiety symptoms. Calcium channel modulators targeting *CACNA1C* are available, but methylation effects are context-dependent (Starnawska et al., 2016; Kabir et al., 2017), and inappropriate modulation may cause neural or cardiovascular off-target effects (Harrison et al., 2022). Functional validation establishing the effects of observed methylation changes on *CACNA1C* expression and activity is essential before therapeutic targeting can be considered.

If the dysbiosis we observed is confirmed to contribute to disease maintenance or complications through longitudinal and mechanistic studies, microbiome-targeted interventions (probiotics, prebiotics, dietary modifications) might benefit certain patients (Cryan et al., 2019), though personalized approaches based on individual microbiome profiles may be necessary.

From a developmental perspective, if AN is associated with disrupted neurodevelopment during adolescent critical periods, early intervention during this high-plasticity window could be important (Paus et al., 2008). However, the effectiveness of such developmentally-timed interventions remains to be empirically tested.

### 4.5 Limitations

Several important limitations must be acknowledged. First, we explicitly state the exploratory nature of this study. Most critically, our cross-sectional design cannot establish causality or temporal relationships. Longitudinal studies examining individuals before illness onset, during acute illness, and after recovery are essential. The use of peripheral tissue (saliva) may not capture brain-specific processes. Moreover, saliva contains heterogeneous cell populations (epithelial cells, immune cells, bacteria), and salivary exosomes derive from multiple cellular sources; variability in cellular composition may confound methylation and miRNA signals, and cell-type deconvolution was not performed due to the lack of validated reference libraries for salivary cell types.

The pooled sample design for RRBS and miRNA prevented individual-level analysis and adjustment for clinical covariates (age, medication, illness duration, AN subtype). While our sample quality control showed no systematic differences and subtype distribution was balanced across pools, we cannot exclude the possibility that subtype-related biological differences (including potential effects of purging behaviors on oral environment) contribute to within-group heterogeneity. Similarly, the inclusion of adolescent AN participants (≥14 years) versus adult controls (≥18 years, due to ethical constraints associated with recruiting healthy minors for research involving biological sampling without direct therapeutic benefit) introduces age-related heterogeneity, reflecting both clinical reality of adolescent AN onset and ethical constraints on recruiting healthy minors. While this may confound our findings, the identification of consistent molecular signatures across both adolescent and adult patients supports the notion that these alterations are stable and long-term consequences of early neurodevelopmental disruption rather than transient developmental drift. Our AN cohort had a mean BMI of 18.7 ± 2.4 (range: 14–24) at the time of saliva collection, reflecting mild-to-moderate illness severity. Findings may not generalize to severely malnourished acute patients, and molecular signatures could differ across BMI severity ranges. We should also acknowledge that our AN cohort included both restrictive (61%) and binge-purge (39%) subtypes. Additionally, the presence of other psychiatric conditions was systematically evaluated in the clinical sample but not in the HC group (recruitment via fliers/advertisement), and a potential effect of undiagnosed disorders on the analysis cannot be formally excluded.

Moreover, we did not measure gene expression levels for any of the genes showing altered methylation levels or perform functional validation to demonstrate how molecular changes affect target gene expression, neuronal function, or behavior. Potential confounding medications, comorbidities, illness duration, and treatment history cannot be fully excluded.

Lastly, for the microbiome findings specifically, while we have excluded technical contamination as the primary explanation, we cannot definitively establish the mechanisms of driving dysbiosis or determine whether it represents cause or consequence of illness. We also lack longitudinal data showing whether dysbiosis precedes illness onset, develops during illness, or persists after recovery.

### 4.6 Future directions

Priority future directions include longitudinal studies tracking molecular changes across illness courses, with particular value in prospective studies examining high-risk individuals before onset (Treasure et al., 2015). Validation of key miRNAs by quantitative PCR in independent cohorts using individual-level samples, rather than pooled specimens, is essential to confirm the trends observed in our pooled analysis. Furthermore, future research must include parallel assessment of methylation and gene expression to establish functional significance, since DNA methylation alterations do not invariably translate to expression changes, and the functional consequences depend on genomic location and cellular context. Functional studies using miRNA mimics or inhibitors, as well as models demonstrating how these molecular changes affect neuronal function, will be necessary to move beyond correlative observations (Issler and Chen, 2015). Regarding microbiome, our findings demonstrate the importance of examining heterogeneity rather than assuming all patients show similar patterns and they highlight the need for detailed clinical characterization to identify factors associated with different microbial profiles. To address these needs, future research should focus on: (1) longitudinal sampling to determine when dysbiosis develops; (2) detailed dietary assessment to identify nutritional factors; (3) functional metagenomic analysis to assess metabolic capabilities of dysbiotic communities; (4) examination of both oral and gut microbiomes; (5) investigation of microbiome metabolites and their potential effects on host physiology; and (6) testing whether microbiome patterns predict treatment outcomes, which would allow microbiome composition to serve as a biomarker for patient stratification and the identification of subgroups with different treatment needs. Finally, these insights raise the possibility that individuals might benefit from microbiome-targeted interventions (probiotics, prebiotics, or dietary modifications), though this will require careful clinical testing. Integration with genetic data through methylation quantitative trait loci (mQTL) and miR-expression quantitative trait loci (miR-eQTL) analysis would distinguish primary molecular changes from those secondary to genetic variation (Aguet et al., 2017). Additionally, the application of long-read sequencing technologies would enable simultaneous capture of sequence variation and methylation data at single-nucleotide resolution, strengthening the mechanistic interpretation reported here. Multi-tissue comparisons would reveal relationships between peripheral and central molecular signatures.

## 5 Conclusion

This integrated multi-omics analysis uncovers a coherent pattern of molecular dysregulation in AN, spanning DNA methylation, miRNA networks, and microbiome composition, collectively pointing to disrupted neurodevelopmental regulation during a critical period of neural circuit maturation. The miR-135 family targeting *KCNN3*, miR-374b affecting *IGFBP3*, and *CACNA1C* methylation alterations collectively suggest coordinated disruption of mechanisms governing neural circuit maturation. The identification of a dysbiotic microbiome subgroup (42% of patients) reveals previously unrecognized disease heterogeneity. These findings support an emerging neurodevelopmental framework for understanding eating disorders (Kaye et al., 2013), with miRNA dysregulation representing the most robust molecular signature. While our study has important limitations—cross-sectional design, peripheral tissue analysis, pooled samples, and lack of functional validation—it provides a foundation for mechanistic studies and generates specific testable hypotheses. The molecular signatures identified here could potentially serve as non-invasive biomarkers, and the pathways represent potential therapeutic targets pending validation. This work demonstrates that multi-layered omics approaches can reveal convergent pathways and disease heterogeneity in eating disorders (Hasin et al., 2017), contributing to biologically-informed, developmentally-sensitive treatment development.

## Data Availability

The raw data supporting the conclusions of this article will be made available by the authors, without undue reservation.
