# Supplementary material for: Dysregulation of neurodevelopmental regulatory networks in Anorexia Nervosa: an integrated multi-layered omics analysis
Source: Front Cell Dev Biol. 2026 May 19;14:1761785. doi: 10.3389/fcell.2026.1761785 (PMC13226574; doi:10.3389/fcell.2026.1761785)
Supplement: Supplementary file 1 [file DataSheet1.docx]

Supplementary Material

**Figures**

**
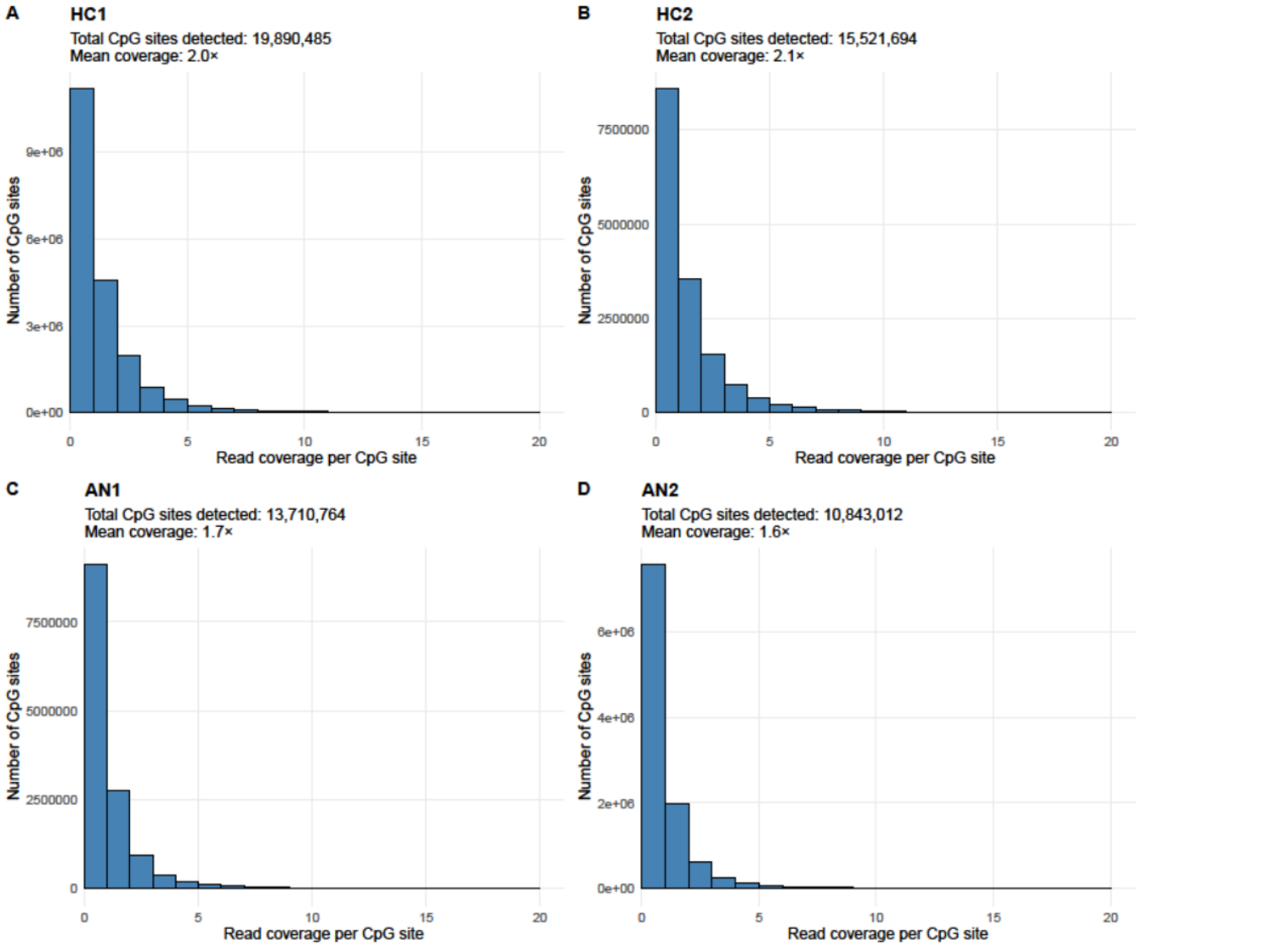
**

**Figure S1.** CpG coverage distribution across pooled libraries within the 0-20 x range**.** AN1, AN2: Anorexia Nervosa groups; HC1, HC2: Healthy Control groups

**Tables**

**Table S1.** Clinical and demographic profiles of the RRBS pooled libraries

|  | **RRBS** | | | |
| --- | --- | --- | --- | --- |
| **Pool** | **HC1** | **HC2** | **AN1** | **AN2** |
| **Characteristics** | Mean *± SD* | Mean *± SD* | Mean *± SD* | Mean *± SD* |
| *n. of individuals* | 10 | 10 | 10 | 10 |
| *Age (years)* | 19.3 ± 0.5 | 19.6 ± 1.0 | 17.7 ± 3.6 | 17.8 ± 4.5 |
| *BMI at saliva collection (kg/m^2^)* | 21.2 ± 1.7 | 20.2 ± 2.3 | 19.1 ± 2.6 | 18.1 ± 2.1 |
| *Age of onset ± SD* | - | - | 16 ± 3.4 | 15.6 ± 3.3 |
| **AN Clinical features** |  |  |  |  |
| *Illness duration (years)* | - | - | 1.7 ± 1.1 | 2.2 ± 2.2 |
| **AN subtype** |  |  |  |  |
| *Restrictive* | - | - | 7 (70%) | 7 (70%) |
| *Binge-purge* | - | - | 3 (30%) | 3 (30%) |
| **Medications** |  |  |  |  |
| *Antidepressants* | - | - | 1 (10%) | 1 (10%) |
| *Antipsychotics* | - | - | 1 (10%) | 1 (10%) |
| *Antidepressants +*  *Antipsychotics* | - | - | 5 (40%) | 4 (40%) |
| *Other* | - | - | 0 (0%) | 0 (0%) |
| *None* | - | - | 3 (30%) | 4 (20%) |

**Table S2.** RRBS metrics across pooled libraries. A: Quality Control summary. B: Distribution of CpG coverage across pooled libraries.

| **A** | **Total sequences** | **Unique alignments** | **Mapping (%)** | **Duplication (%)** | **Bisulfite conversion (%)** | **CpG methylation level (%)** |
| --- | --- | --- | --- | --- | --- | --- |
| HC1 | 68617449 | 29159675 | 42.5 | 24.06% | 99.78 | 78.25 |
| HC2 | 64329985 | 24679016 | 38.36 | 23.89% | 99.76 | 78.66 |
| AN1 | 42670544 | 23185559 | 54.34 | 20.63% | 99.77 | 80.83 |
| AN2 | 41754110 | 14561531 | 34.87 | 26.90% | 99.75 | 77.54 |

| **B** | **N total sites** | **% coverage ≥ 1x** | **% coverage ≥ 5x** | **% coverage ≥ 10x** | **% coverage ≥ 20x** | **Mean coverage** | **Median coverage** |
| --- | --- | --- | --- | --- | --- | --- | --- |
| HC1 | 19890485 | 100 | 6.35 | 1.14 | 0.13 | 2.02 | 1 |
| HC2 | 15521694 | 100 | 6.93 | 1.27 | 0.14 | 2.09 | 1 |
| AN1 | 13710764 | 100 | 4.01 | 0.73 | 0.07 | 1.71 | 1 |
| AN2 | 10843012 | 100 | 3.65 | 0.71 | 0.08 | 1.64 | 1 |

**Table S3.** Clinical and demographic profiles of the miRNA pooled libraries

|  | **miRNome** | | | | | | | | |
| --- | --- | --- | --- | --- | --- | --- | --- | --- | --- |
| **Pool** | **HC1** | **HC2** | **HC3** | **HC4** | **HC5** | **HC6** | **AN1** | **AN2** | **AN3** |
| **Characteristics** | Mean *± SD* | Mean *± SD* | Mean *± SD* | Mean *± SD* | Mean *± SD* | Mean *± SD* | Mean *± SD* | Mean *± SD* | Mean *± SD* |
| *n. of individuals* | 5 | 10 | 7 | 5 | 5 | 4 | 10 | 10 | 10 |
| *Age (years)* | 19.2 ± 0.4 | 19.4 ± 0.5 | 19.3 ± 0.5 | 20 ± 1 | 19 ± 0.7 | 19.8 ± 0.5 | 18.5 ± 4.6 | 17.4 ± 2.9 | 19.9 ± 6.6 |
| *BMI at saliva collection (kg/m^2^)* | 21.8 ± 0.8 | 20.8 ± 1.0 | 23.0 ± 2.2 | 20.3 ± 2.7 | 19.5 ± 2.2 | 21.3 ± 2.4 | 18.1 ± 2.0 | 19.6 ± 1.7 | 17.8 ± 2.4 |
| *Age of onset ± SD* | - | - | - | - | - | - | 16.1 ± 3.5 | 15.4 ± 2.5 | 16.1 ± 4.7 |
| **AN Clinical features** |  |  |  |  |  |  |  |  |  |
| *Illness duration (years)* | - | - | - | - | - | - | 2.4 ± 2.5 | 2.0 ± 1.2 | 3.8 ± 3.8 |
| **AN subtype** |  |  |  |  |  |  |  |  |  |
| *Restrictive* | - | - | - | - | - | - | 8 (80%) | 6 (60%) | 6 (60%) |
| *Binge-purge* | - | - | - | - | - | - | 2 (20%) | 4 (40%) | 4 (40%) |
| **Medications** |  |  |  |  |  |  |  |  |  |
| *Antidepressants* | - | - | - | - | - | - | 1 (10%) | 0 (10%) | 1 (10%) |
| *Antipsychotics* | - | - | - | - | - | - | 0 (0%) | 0 (0%) | 2 (20%) |
| *Antidepressants +*  *Antipsychotics* | - | - | - | - | - | - | 6 (50%) | 7 (70%) | 2 (20%) |
| *Other* | - | - | - | - | - | - | 0 (0%) | 0 (0%) | 0 (0%) |
| *None* | - | - | - | - | - | - | 3 (30%) | 3 (30%) | 5 (40%) |

**Table S4.** Differentially methylated CpG sites in protein-coding genes

| **chr. N** | **pos.** | **total coverage** | **HC** | **AN** | **mean meth. difference (Δβ) AN vs HC** | **annotation types** | **refseq ids** | **GENE** |
| --- | --- | --- | --- | --- | --- | --- | --- | --- |
|  |  |  | **Meth %** | **Meth %** |  |  |  |  |
| chr1 | 634166 | 48 | 25.00 ± 7.07 | 0.00 ± 0.00 | -0.25 | promoter | NM_001005277 | *OR4F3* |
| chr1 | 634170 | 44 | 29.46 ± 11.36 | 0.00 ± 0.00 | -0.30 | promoter | NM_001005277 | *OR4F3* |
| chr1 | 634178 | 42 | 18.75 ± 8.84 | 0.00 ± 0.00 | -0.19 | promoter | NM_001005277 | *OR4F3* |
| chr11 | 65116960 | 29 | 15.00 ± 7.07 | 0.00 ± 0.00 | -0.15 | promoter | NM_014205 | *ZNHIT2* |
| chr12 | 2256000 | 36 | 86.19 ± 0.67 | 100.00 ± 0.00 | 0.14 | intron | NM_000719 | *CACNA1C* |
| chr18 | 108223 | 580 | 60.69 ± 4.81 | 35.42 ± 2.95 | -0.25 | promoter | NR_033770 | *ROCK1* |
| chr21 | 10419455 | 52 | 79.50 ± 6.36 | 60.00 ± 0.00 | -0.20 | intron | NM_182482 | *BAGE2* |
| chr21 | 10463400 | 30 | 36.25 ± 33.59 | 90.00 ± 14.14 | 0.54 | intron | NM_182482 | *BAGE2* |
| chr21 | 10478652 | 64 | 3.57 ± 5.05 | 100.00 ± 0.00 | 0.96 | intron | NM_182482 | *BAGE2* |
| chr3 | 196898846 | 27 | 80.56 ± 3.93 | 100.00 ± 0.00 | 0.19 | intron | NM_001308045 | *SENP5* |
| chrX | 49590327 | 29 | 83.77 ± 2.75 | 100.00 ± 0.00 | 0.16 | intron | NM_001127212 | *GAGE2A* |

**Table S5.** Differentially expressed miRNAs in AN patients versus controls*.* miRNA ID indicates the specific miRNA name, the log2 fold change (log2FC) represents the magnitude of differential ecpression, and the direction of regulation specifies whether the miRNA is up‑regulated (UP) or down‑regulated (DOWN).

| **miRNA ID** | **log2FC** | **Direction** |
| --- | --- | --- |
| hsa-miR-136-5p | 4.51 | UP |
| hsa-miR-31-3p | 3.17 | UP |
| hsa-miR-524-3p | 2.92 | UP |
| hsa-miR-550a-5p | 4.11 | UP |
| hsa-miR-532-5p | 4.29 | UP |
| hsa-miR-765 | 2.22 | UP |
| hsa-miR-133b | 2.32 | UP |
| hsa-miR-589-5p | 2.42 | UP |
| hsa-miR-141-3p | 2.34 | UP |
| hsa-miR-934 | 2.16 | UP |
| hsa-miR-200a-3p | 2.31 | UP |
| hsa-miR-99b-5p | 2.23 | UP |
| hsa-miR-23b-3p | 2.05 | UP |
| hsa-miR-500a-5p | 3.65 | UP |
| hsa-miR-887-3p | 2.03 | UP |
| hsa-miR-625-3p | 3.00 | UP |
| hsa-miR-181d-5p | 5.03 | UP |
| hsa-miR-374b-5p | 2.28 | UP |
| hsa-miR-151a-3p | 2.08 | UP |
| hsa-miR-202-3p | 4.12 | UP |
| hsa-miR-362-5p | 2.69 | UP |
| hsa-miR-363-3p | 2.44 | UP |
| hsa-miR-132-3p | 2.11 | UP |
| hsa-miR-187-3p | 4.74 | UP |
| hsa-miR-548b-3p | 3.03 | UP |
| hsa-miR-485-3p | 2.04 | UP |
| hsa-miR-200b-3p | 2.60 | UP |
| hsa-miR-637 | 2.71 | UP |
| hsa-miR-34c-5p | 7.27 | UP |
| hsa-let-7f-5p | 3.63 | UP |
| hsa-miR-215-5p | 2.41 | UP |
| hsa-miR-30b-5p | 2.05 | UP |
| hsa-miR-335-5p | 2.52 | UP |
| hsa-miR-214-3p | 5.45 | UP |
| hsa-miR-371a-5p | 3.25 | UP |
| hsa-miR-608 | 2.00 | UP |
| hsa-miR-671-5p | 2.28 | UP |
| hsa-miR-152-3p | 2.36 | UP |
| hsa-miR-124-3p | 4.31 | UP |
| hsa-miR-595 | 3.13 | UP |
| hsa-miR-602 | 2.16 | UP |
| hsa-miR-376a-3p | 3.84 | UP |
| hsa-miR-449a | 7.23 | UP |
| hsa-miR-135b-5p | 3.36 | UP |
| hsa-miR-744-5p | 3.36 | UP |
| hsa-miR-18a-3p | 2.11 | UP |
| hsa-miR-9-5p | 5.76 | UP |
| hsa-miR-134-5p | 3.15 | UP |
| hsa-miR-200c-3p | 2.83 | UP |
| hsa-miR-30a-5p | 2.98 | UP |
| hsa-miR-874-3p | 2.18 | UP |
| hsa-miR-135a-5p | 4.31 | UP |
| hsa-miR-182-5p | 2.01 | UP |
| hsa-miR-411-5p | 2.87 | UP |
| hsa-miR-216b-5p | 3.16 | UP |
| hsa-miR-193a-3p | 3.82 | UP |
| hsa-let-7e-5p | 2.54 | UP |
| hsa-miR-100-5p | 6.09 | UP |
| hsa-miR-629-5p | 2.44 | UP |
| hsa-miR-429 | 2.00 | UP |
| hsa-miR-373-5p | 2.15 | UP |
| hsa-miR-642a-5p | 3.90 | UP |
| hsa-miR-31-5p | 2.05 | UP |
| hsa-miR-570-3p | -3.06 | DOWN |
| hsa-miR-545-3p | -2.96 | DOWN |
| hsa-miR-665 | -2.08 | DOWN |
| hsa-miR-154-3p | -2.69 | DOWN |
| hsa-miR-22-5p | -2.41 | DOWN |
| hsa-miR-19a-3p | -2.16 | DOWN |
| hsa-miR-133a-3p | -2.69 | DOWN |
| hsa-miR-410-3p | -2.26 | DOWN |
| hsa-miR-520d-5p | -3.13 | DOWN |
| hsa-miR-195-5p | -2.22 | DOWN |
| hsa-miR-518a-3p | -2.71 | DOWN |

**Table S6.** PERMANOVA Pseudo-F (999 permutations) test results on unweighted unifrac distances for beta diversity evaluation

| **REFERENCE** | **Group 2** | **Sample size** | **Pseudo-F** | **p-value/**  **q-value** |
| --- | --- | --- | --- | --- |
| HC | AN-sub | 54 | 7.94 | 0.001 |
| HC | AN | 59 | 2.58 | 0.001 |
| AN | AN-sub | 33 | 3.68 | 0.001 |

**Table S7.** Alpha diversity analysis: Kruskal-Wallis test results for Faith phylogenetic diversity (PD) and Evenness vector distances

|  | **H PD** | **p-value PD** | **H Evenness** | **p-value Evenness** |
| --- | --- | --- | --- | --- |
| All-groups | 22.42 | 0.0000135 *** | 42.42 | 6.14*10^-10 *** |
| AN-sub vs HC | 18.178 | 0.000020 *** | 30.55 | 3.26*10^-8 *** |
| AN vs HC | 7.696 | 0.0055 ** | 14.96 | 1.13*10^-4 *** |
| AN vs AN-sub | 6.317 | 0.011958 | 20.73 | 5.28*10^-6 *** |
